# Long-term prediction of algal chlorophyll based on empirical models and the machine learning approach in relation to trophic variation in Juam Reservoir, Korea

**DOI:** 10.1016/j.heliyon.2024.e31643

**Published:** 2024-05-28

**Authors:** Sang-Hyeon Jin, Namsrai Jargal, Thet Thet Khaing, Min Jae Cho, Hyeji Choi, Bilguun Ariunbold, Mnyagatwa Geofrey Donat, Haechan Yoo, Md Mamun, Kwang-Guk An

**Affiliations:** a Department of Bioscience and Biotechnology, Chungnam National University, Daejeon, 34134, Republic of Korea; b Department of Earth Sciences, Southern Methodist University, Dallas, TX, 75205, USA

**Keywords:** Algal chlorophyll, Empirical analysis, Nutrient regimes, Machine learning, Summer monsoon, Temperate reservoir

## Abstract

This study analyzed spatiotemporal variation and long-term trends in water quality indicators and trophic state conditions in an Asian temperate reservoir, Juam Reservoir (JR), and developed models that forecast algal chlorophyll (CHL-a) over a period of 30 years, 1993–2022. The analysis revealed that there were longitudinal gradients in water quality indicators along the reservoir, with notable influences from tributaries and seasonal variations in nutrient regimes and suspended solids. The empirical model showed phosphorus was found to be the key determinant of algal biomass, while suspended solids played a significant role in regulating water transparency. The trophic state indices indicated varying levels of trophic status, ranging from mesotrophic to eutrophic. Eutrophic states were particularly observed in zones after the summer monsoons, indicating a heightened risk of algal blooms, which were more prevalent in flood years. The analysis of trophic state index deviation suggested that phosphorus availability strongly influences the reservoir trophic status, with several episodes of non-algal turbidity at each site during Mon. Increases in non-algal turbidity were more prevalent during the monsoon in flood years. This study also highlighted overall long-term trends in certain water quality parameters, albeit with indications of shifting pollution sources towards non-biodegradable organic matter. According to the machine learning tests, a random forest (RF) model strongly predicted CHL-a (R^2^ = 0.72, p < 0.01), except for algal biomass peaks (>60 μg/L), compared to all other models. Overall, our research suggests that CHL-a and trophic variation are primarily regulated by the monsoon intensity and predicted well by the machine learning RF model.

## 1 Introduction

Reservoirs are crucial freshwater sources because they serve multiple functions, including drinking water supply, hydropower generation, flood control, and recreational activities [1]. However, human activities such as industrialization, urbanization, and intensive agriculture in catchment areas have led to disturbances impacting the water quality of lentic ecosystems like lakes and reservoirs [2]. Among the challenges these systems face, nutrient enrichment stands out, resulting in eutrophication and diminished water quality [3]. Both anthropogenic discharges and internal processes contribute to this issue, fostering excessive algal growth [4,5]. Hence, monitoring water quality in artificial reservoirs becomes imperative to comprehend nutrient dynamics and accurately predict algal biomass.

Eutrophication management primarily relies on empirical models that establish the relationship between nutrients (nitrogen and phosphorus (P)) and algal chlorophyll (CHL-a) levels [6,7]. While some models focus on individual lentic systems [[8], [9], [10]], others encompass broader datasets covering numerous lakes at national and regional levels [7,11,12]. The positive relation between nutrients and CHL-a underscores the significance of P-loading, mainly associated with anthropogenic pollution sources, to mitigate excessive algal biomass and manage eutrophication [4,6,13]. However, empirical relationships between P and CHL-a can vary due to geographical location, land use, reservoir morphology, and climatic region [[14], [15], [16], [17], [18]], as well as local moderating factors such as light availability, grazing pressure, water temperature (WT), and water transparency [[19], [20], [21], [22], [23]].

Trophic state classification is fundamental for assessing reservoir health and eutrophication impact on water quality. Various concentration criteria and trophic state indices are employed for this purpose at regional and global scales [24,25]. The Trophic State Index (TSI) by Carlson [24] stands out as one of the widely used methods, utilizing parameters like total P (TP), CHL-a, and Secchi depth (SD) to categorize reservoirs into oligotrophic, mesotrophic, eutrophic, and hypereutrophic states. These classifications are pivotal for developing predictive eutrophication models to curb algal blooms and identify causative factors. Also, a two-dimensional graphical approach based on the trophic state index deviation (TSID) can provide important insight into the spatial and seasonal changes in nutrient levels, light availability, and zooplankton grazing that potentially influence algal growth seasonally and spatially [19].

In regions experiencing seasonal monsoons, rainfall intensity significantly influences water quality and algal growth, primarily through variations in inflow rates and hydraulic retention time (HRT) [8,26,27]. South Korea experiences summer monsoons, mainly from July to August [14]. It encounters substantial changes in nutrient levels, suspended solids (SS), light availability, and algal CHL-a in reservoirs due to pollutant-rich runoff and sediment resuspension [11,28,29]. These seasonal fluctuations, including rapid flushing and dilution, can alter algal growth, challenging the predictive accuracy of statistical models [23,26,30].

The complexity of environmental factors can diminish the predictive accuracy of algal biomass models for individual systems based on a single factor, such as key nutrients [30,31]. Machine-learning approaches are increasingly used to predict algal growth in lentic systems because they can provide insights into the importance of input variables and their interactions for predicting algal CHL-a [30,32,33]. Random forest (RF), support vector machine (SVM), and artificial neural network (ANN) models have been applied to predict CHL-a levels, blue-green algal blooms, and water transparency in lacustrine systems globally [[32], [33], [34], [35], [36]]. However, each machine learning algorithm has its own advantages and disadvantages. Therefore, selecting the most effective algorithm or combination of algorithms for predicting algal biomass in lentic systems may be challenging. Huang et al. [37] predicted algal CHL-a from environmental factors in Chinese lentic systems using RF and a generalized linear model. They found TP was the main factor influencing CHL-a. Meanwhile, Mamun et al. [30] utilized multiple linear regression (MLR), SVM, and ANN to predict algal CHL-a and SD in a monsoon-region reservoir and found that SVM performed better than MLR and ANN.

The Juam Reservoir (JR), located within a monsoonal climate region, is a pivotal water resource catering to various societal needs, including water supply, irrigation, and recreational activities. Despite the absence of substantial water quality problems, previous research endeavors have shed light on the hydrological conditions, water quality dynamics, and nutrient loading patterns within the reservoir's watershed. Han et al. [38] identified phosphorus as a critical factor driving eutrophication in JR, while Yi et al. [39] assessed hazardous sub-watersheds contributing to non-point source pollution, providing valuable insights for targeted management strategies. The study of [40] comprehensively monitored water quantity and quality in an upstream tributary of JR, highlighting the significant influence of land use practices on nutrient loading. Furthermore, Chung et al. [41] elucidated the relationship between harmful cyanobacteria and environmental factors in JR, underscoring the impact of runoff-induced phosphorus inputs on algal biomass dynamics during rainfall events.

This study, based on 30 years of monthly data for JR, comprehensively analyzed the longitudinal-seasonal dynamics of physicochemical indicators of water quality, CHL-a, and trophic state conditions to identify critical factors determining algal biomass and water transparency with the help of multivariate analysis and empirical linear models. We also compared the differences in water quality indicators, TSI, and TSID between drought and flood year regimes, depending on the intensity of the summer monsoon. Furthermore, we assessed overall long-term trends of water physicochemical indicators and CHL-a using the Mann-Kendall test and innovative trend analysis and tested the machine learning technique for long-term CHL-a and SD prediction models. Overall, this study would provide important insights into the factors influencing water quality and CHL-a dynamics in the JR and help future decision-making for sustainable water supplies and ecological health.

## 2 Materials and methods

### 2.1 Study system

**Fig. 1:**
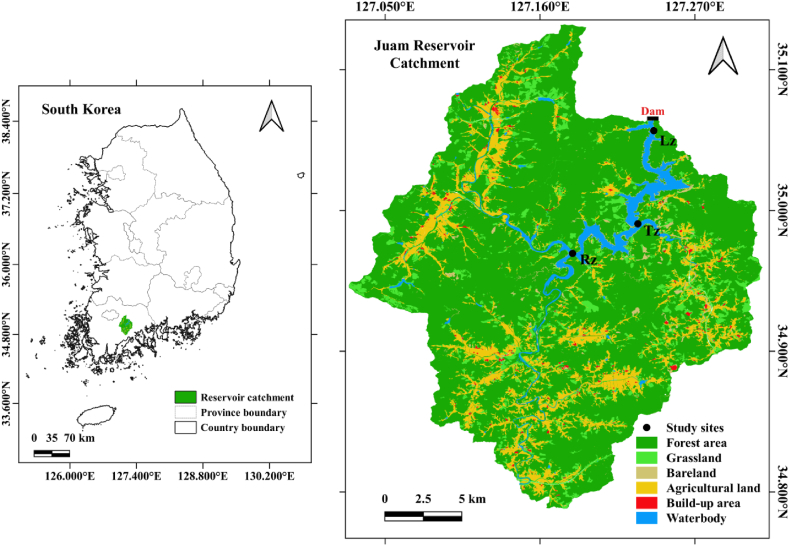
Location of Juam Reservoir and the study sites, including lacustrine (Lz), transition (Tz), and riverine (Rz) zones, with land cover and land use patterns.

The JR is an artificial lake that was initiated in 1992 through the construction of a multi-purpose dam on the Seomjin River in South Korea (Fig. 1). It is a warm monomictic reservoir that is about 40 km long and has a surface area of 33 square kilometers, with an average depth of 14 m, maximum depth of 47 m, and basin area of 1010 square kilometers. It is a critical freshwater source for the region, serving the water needs of Gwangju, Naju, and Mokpo cities by supplying 25 million cubic meters of drinking water daily [32]. The reservoir catchment area mainly consists of forested mountains (70.5 %), with agriculture (13.2 %) and built-up areas (1.5 %) as the primary land uses (Fig. 1). Non-point pollution sources are key managing issues for JR due to high nutrient loadings released from agricultural lands during summer monsoons [38,39]. In this study, we used long-term data from three study sites representing the longitudinal zones of the reservoir: Rz (riverine zone), Tz (transition zone), and Lz (lacustrine zone) (Fig. 1).

### 2.2 Data sources and water quality analysis

The physicochemical data used in this study were obtained from the Korean Water Environment Information System (https://water.nier.go.kr/), managed by the Korea Ministry of Environment (MOE). The analysis of the monsoon season was conducted by taking samples during three distinct periods - the premonsoon period (Pre), which spans from May to June; the monsoon period (Mon), which occurs during July and August; and the postmonsoon period (Post), which lasts from September to October. Thirteen physicochemical variables were studied: WT, electrical conductivity (EC), SS, biological oxygen demand (BOD), chemical oxygen demand (COD), total organic carbon (TOC), total nitrogen (TN), ammonium-nitrogen (NH4–N), nitrate-nitrogen (NO_3_–N), TP, phosphate-phosphorus (PO_4_–P), algal CHL-a, and SD. The study period was 1993–2022, and monthly data were collected from the water surface layer (at 0.5 m depth) at each site. Monthly data on water level and precipitation were obtained from the Water Information Portal (https://www.water.or.kr), managed by the Korea Water Resources Corporation (K-water).

WT and EC were measured on-site using a YSI Sonde 6600 multi-parameter sensor (Environmental Monitoring Systems, Yellow Springs, OH, USA). SD was measured on-site using a standard 20-cm Secchi disk as water transparency indicator. Determination of the levels of organic matter (BOD, COD, and TOC), SS, nutrients, and CHL-a was followed by a standard procedure of MOE [42]. SS was determined in samples filtered through a 1.2-μm pore size glass microfiber filter (grade GF/C; Whatman) and dried for 1 h at 105 °C [43]. COD was determined by oxidizing the water sample with potassium permanganate at 100 °C for 30 min after acidifying it with sulfuric acid [42,44]. A second derivative procedure was applied for TN after the digestion of persulfate [45], while ascorbic acid was used after oxidation to determine TP [46]. To measure CHL-a concentrations, the water sample was passed through a GF/C filter and extracted in ethanol before being assessed spectrophotometrically (DU-530; Beckman Coulter, Brea, CA, USA).

### 2.3 Statistical analysis

#### 2.3.1 Empirical regression models and principal component analysis

The data were first log_10_-transformed to address normality and heteroscedasticity concerns. The transformed spatial-seasonal averages of the designated variables were then used for empirical linear models (EMs), multiple linear regression (MLR), and principal component analysis (PCA). The regression analyses were performed using SigmaPlot v14.5 (Systat, Santa Clara, CA, USA), and PCA was conducted using PAST v4.12 (University of Oslo, Norway).

#### 2.3.2 Long-term trend analysis

We conducted a long-term trend analysis to detect significant trends in yearly mean values of water quality indicators in the reservoir from 1993 to 2022. The Mann–Kendall (MK) test and innovative trend analysis (ITA) were applied for this purpose.

The MK test is a non-parametric statistical method [47,48]. It checks the null hypothesis that there is no trend in the data against the alternative hypothesis that there is a trend. The following steps are carried out to perform the MK test. The formula for the MK test statistic S is:

$$S=\sum_{i=1}^{n-1}\sum_{j=i+1}^{n}sgn\left(x_{j}-x_{i}\right)$$

$$sgn\left(x_{j}-x_{i}\right)=\left\{ \begin{array}{c} 1\;\left(\text{if}\;x_{j}-x_{i}>0\right)\\ 0\;\left(\text{if}\;x_{j}-x_{i}=0\right)\\ -1\;\left(\text{if}\;x_{j}-x_{i}<0\right) \end{array}\right.$$

The variance is computed as:

$$Var\left(S\right)=\frac{n\left(n-1\right)\left(2n+5\right)-\sum_{i=1}^{m}t_{i}\left(t_{i}-1\right)\left(2t_{i}+5\right)\;}{18}$$

Then, the standard normal test statistic is calculated as:

$$Z=\left\{ \begin{array}{c} \frac{S-1}{\sqrt{Var\left(S\right)}}\;\left(\text{if}\;S>0\right)\\ \;0\;\left(\text{if}\;S=0\right)\;\\ \frac{S+1}{\sqrt{Var\left(S\right)}}\;\left(\text{if}\;S<0\right) \end{array}\right.$$

Sen's slope is a non-parametric method used to estimate the magnitude and direction of the trend in time series data [49] and is defined as:

$$Sen^{\prime }s\;slope=Median\left\{\frac{x_{j}-x_{i}}{j-i}:i<j\right\}$$

for the set of pairs (i, xi) where xi is time series data. The slope represents the rate of change of the data over time and can be used to estimate the magnitude and direction of the trend. These methods have been widely used to detect trends in hydrological time series data because they can extract useful information on the possibility of future changes in water quality variables. Moreover, no assumptions about the distribution of the data must be met [[50], [51], [52]]. We performed these tests using the “*mannKen*” function in the R package “*wql*”, a statistical programming language used for data analysis.

The ITA is used to identify deterministic trends in observed time series [53]. It can be used without assumptions, distinguishing it from classical approaches like the MK trend test and Spearman's rho, which typically impose certain assumptions. The method begins by dividing the time series into two equal parts, which are then sorted in ascending order. The x-axis represents the first half of the time series, while the y-axis represents the second half. When the data points align along the ideal 1:1 line, it indicates the absence of a trend in the time series. However, if the data points accumulate in the region above the ideal line, it suggests the presence of an increasing trend in the time series. Conversely, if the data points are below the 1:1 line, it indicates a decreasing trend in the time series [53,54]. We implemented ITA using the “innovtrend” function in the R package “trendchange” and the 10 % confidence level was applied to ensure accurate analysis.

#### 2.3.3 TSI and TSID

TSI values were calculated individually from CHL-a, TP, and SD according to the following formulas [24]:

TSI(TP,μg/L)=10×[6−ln(48/TP)/ln2]

TSI(CHL−a,μg/L)=10×[6−(2.04−0.68ln(CHL−a))/ln2]

TSI(SD,m)=10×[6−ln(SD)/ln2]

The TSI (CHL-a) values were then compared with the TSI (TP) and TSI (SD) values, and the resulting deviations were determined. Two-dimensional plots were then constructed based on these deviations. The degree of P limitation can be inferred from the TSI (CHL-a) – TSI (TP) relationship deviations, while deviations in TSI (CHL-a) and TSI (SD) represent the level of underwater light availability relative to sestonic particles [19]. The two-dimensional plots of TSID were constructed using SigmaPlot v14.5.

#### 2.3.4 Machine learning predictions of algal biomass and water transparency based on RF

In addition to EMs and MLR models, we employed the random forest (RF) machine-learning technique to predict the long-term variations in algal CHL-a and SD levels by assessing by assessing and simulating environmental variables. The RF algorithm is a non-parametric machine learning model that randomly selects subsets of weak predictors and builds shallow regression trees from the training set [55]. The algorithm then aggregates the predictions of all the trees to generate the final output. It is a powerful tool with self-adaptability, self-organization, and error tolerance, and can minimize risk and the upper limit of generalization while also enhancing the generalization ability [37,56,57].

## 3 Results and discussion

### 3.1 Longitudinal variation in water quality and the effect of monsoon season

The data monitoring conducted over a long-term period revealed a longitudinal gradient in water quality indicators along the reservoir (Table 1). The averages of various indicators such as WT, EC, phosphorus contents, SS, and CHL-a increased from the Lz to the Rz, while TN:TP ratio and SD showed decreasing patterns. Studies have found that reservoir systems exhibit a noticeable change in water quality parameters as one moves from the headwaters to the dam zone [10,[58], [59], [60]]. Such changes include variations in suspended solids, nutrients, and primary productivity, which could be due to the morpho-hydrological conditions of the reservoirs. As conditions shift from river-type to lake-type over the longitudinal dimension of the reservoirs, changes in flow velocity, hydraulic retention time, and water depth could cause these variations [8,61]. In addition, external inputs from tributaries could also impact water quality indicators, especially nutrient regimes, inorganic suspended solids, and water transparency. As tributaries carry pollutants, mainly from intensive non-point pollution sources [[38], [39], [40]], into the riverine zone, the water quality can be significantly impacted, leading to higher levels of algal biomass, as measured by CHL-a, in the zones compared to other zones (Table 1). Overall, the temperate reservoir showed low organic matter and a low to moderate state of phosphorus contents and CHL-a, according to Ref. [25].

**Table 1:** The measured water quality parameters along the longitudinal zones (Lz – Lacustrine zone, Tz – Transition zone, Rz – Riverine zone) from 1993 to 2022 (WT: water temperature, EC: electrical conductivity, TN: total nitrogen, NH_4_–N: ammonium-nitrogen, NO_3_–N: nitrate nitrogen, TP: total phosphorusPO_4_-P: Orthophosphate, TN:TP: TN, TP ratio, COD: chemical oxygen demand, BOD: biological oxygen demand, CHL-a: chlorophyll-a, SS: suspended solids, SD: Secchi depth).

Water Quality Parameter		Lz	Tz	Rz	Overall
		Mean ± SD (Min-Max)	Mean ± SD (Min-Max)	Mean ± SD (Min-Max)	Mean ± SD (Min-Max)
WT/ionic indicators	WT (°C)	12.07 ± 4.86 (2–25)	13.78 ± 6.11 (2–26)	15.48 ± 7.55 (0–29)	13.77 ± 6.42 (0–29)
	EC (μS/cm)	75.53 ± 38.03 (35–220)	77.37 ± 26.03 (37–223)	82.24 ± 29.99 (36–265)	78.38 ± 31.87 (35–265)
Organic matter pollution indicators	BOD (mg/L)	0.99 ± 0.40 (0.4–3.8)	1.27 ± 0.43 (0.4–2.7)	1.45 ± 0.46 (0.4–4)	1.24 ± 0.47 (0.4–4)
	COD (mg/L)	2.86 ± 0.53 (1.5–6.7)	2.63 ± 0.38 (1.3–4.3)	2.78 ± 0.48 (1.4–6.0)	2.76 ± 0.48 (1.3–6.7)
	TOC (mg/L)	2.05 ± 0.42 (0.9–3.3)	1.71 ± 0.22 (1.2–2.3)	1.85 ± 0.3 (1.2–2.8)	1.89 ± 0.37 (0.9–3.3)
Nutrient pollution indicators	TN (mg/L)	0.78 ± 0.18 (0.32–1.91)	0.89 ± 0.28 (0.29–2.04)	0.96 ± 0.39 (0.29–2.75)	0.87 ± 0.31 (0.29–2.75)
	NH_4_–N (mg/L)	0.05 ± 0.05 (0–0.28)	0.04 ± 0.05 (0–0.36)	0.05 ± 0.07 (0–0.68)	0.05 ± 0.06 (0–0.68)
	NO_3_–N (mg/L)	0.48 ± 0.14 (0.00–1.24)	0.53 ± 0.18 (0–1.25)	0.56 ± 0.24 (0–1.48)	0.52 ± 0.19 (0–1.48)
	TP (μg/L)	14.25 ± 7.10 (0–54)	17.48 ± 8.62 (4–61)	22.17 ± 11.89 (6–98)	17.96 ± 9.96 (0–98)
	PO_4_–P (μg/L)	2.41 ± 2.09 (0–11)	3.71 ± 3.11 (0–21)	5.07 ± 5.34 (0–57)	3.75 ± 3.93 (0–57)
	TN:TP	69.8 ± 61.1 (14.8–212.2)	59.9 ± 32.8 (8.7–277)	52.1 ± 32.8 (6.3–270.1)	60.61 ± 44.9 (6.3–277)
Algal bloom and transparency indicators	CHL-a (μg/L)	4.29 ± 10.42 (0.10–60.2)	6.12 ± 7.34 (0.10–64.5)	9.13 ± 10.62 (0.3–77.6)	6.52 ± 9.78 (0.1–77.6)
	SS (mg/L)	2.11 ± 1.06 (0.5–11.7)	2.62 ± 1.72 (0.3–11.3)	3.57 ± 3.34 (0.3–31.4)	2.76 ± 2.33 (0.3–31.4)
	SD (m)	3.35 ± 0.91 (0.9–6.1)	2.81 ± 64.5 (1–5.5)	2.38 ± 0.87 (0.5–5.9)	2.84 ± 0.96 (0.5–6.1)

**Fig. 2:**
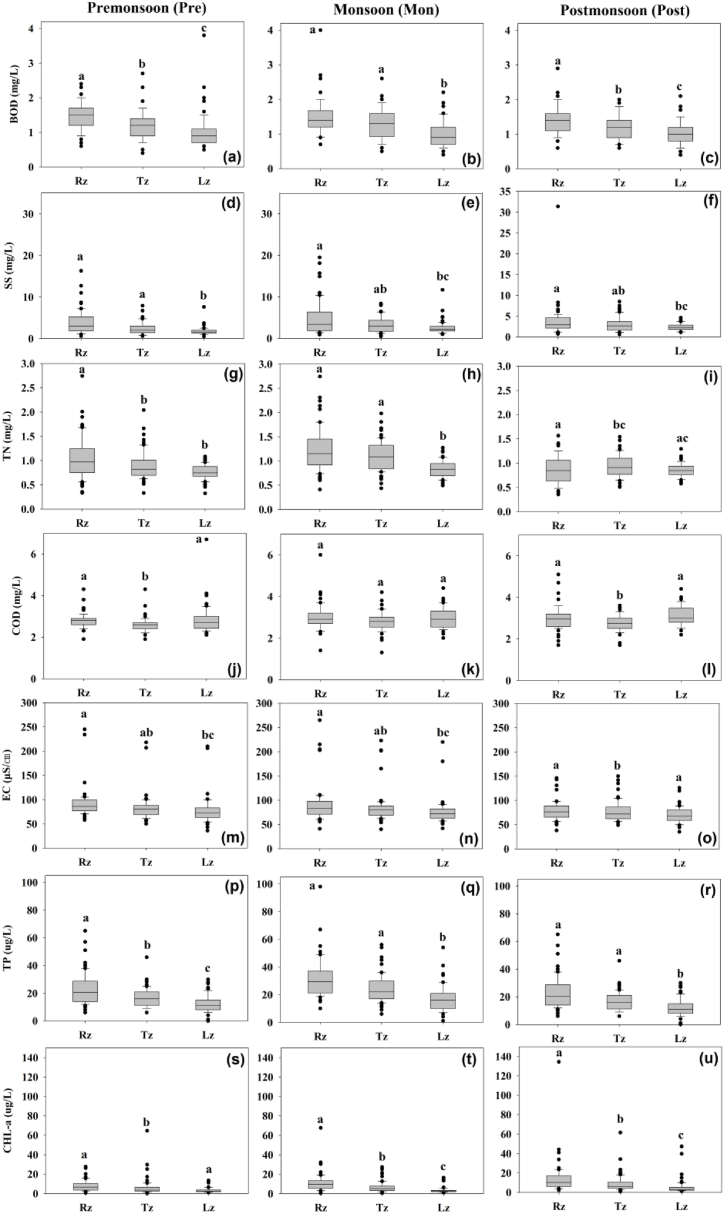
Seasonal variations of water quality and Tukey test at each site (Rz: Riverine zone, Tz: Transition zone, Lz: Lacustrine zone, BOD: biological oxygen demand (a–c), SS: suspended solids (d–f), TN: total nitrogen (g–i), COD: chemical oxygen demand (j–l), EC: electrical conductivity (m–o), TP: total phosphorus (p–r), CHL-a: chlorophyll-a (s–u), the first highest mean receives the letter “a”, and the second and third highest mean receives the letter “b”, and “c”, respectively, and means with no significant difference receive the same letter).

The longitudinal variations in most of the indicators further showed seasonality (Fig. 2a–u). Their means and outliers increased during the Mon in each zone, particularly in terms of suspended solids (Fig. 2d–f) and phosphorus contents (Fig. 2p–r). The elevated levels of SS and TP observed during the Mon generally accorded with increased precipitation and inflow volumes. These findings suggest that there was a greater amount of runoff and phosphorus loading during high rainfall periods due to monsoon [8,38,39]. However, CHL-a levels increased at each site in the Post (Fig. 2s–u). Monsoon-induced nutrient availability and hydrologically stable conditions after the monsoon period could be attributable to the increase in CHL-a level [26,27,31].

### 3.2 Seasonal variation in water quality parameters in drought and flood years

**Fig. 3:**
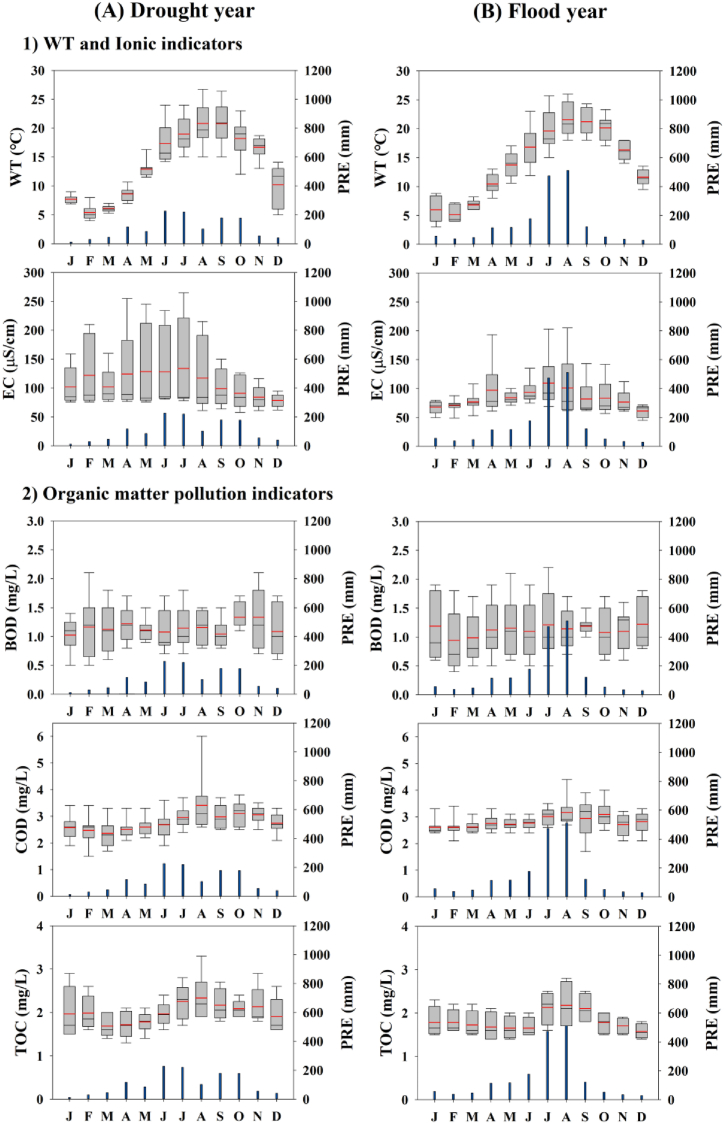
Monthly dynamics in water temperature, the ionic indicator, and organic matter differed between drought (A) and flood years (B) in the reservoir (PRE-precipitation).

**Fig. 4:**
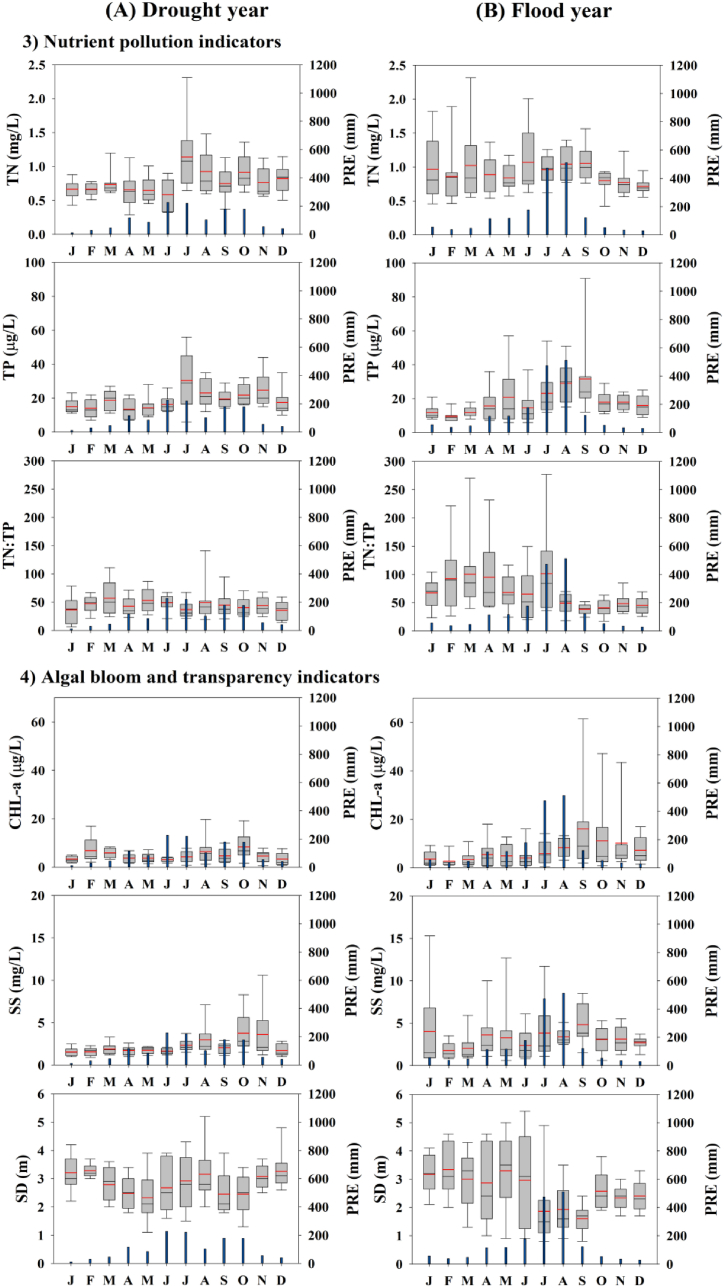
Monthly dynamics in nutrients, TN:TP ratio, algal bloom, and transparency indicators differed between drought (A) and flood years (B) (PRE: precipitation).

Eleven water quality indicators in drought (1996, 2016, and 2019) and flood (2002, 2009, and 2020) years were analyzed to determine the effects of monsoon rainfall on water quality (Fig. 3, Fig. 4). Our analysis indicated that the summer monsoon's intensity significantly influenced the reservoir's seasonal nutrient regimes, suspended solids, water transparency, and algal biomass. By comparing the patterns between years of drought and flood, we observed that the factors mentioned above were affected differently during each type of year.

There was no difference in WT between drought (=3–26) and flood (=4–26.7) years. The difference in EC between the Pre and Mon periods was small. The organic matter pollution indicators (COD and TOC) increased during the summer Mon, consistent with previous studies of Korean reservoirs [10,62,63]. Little difference in organic matter was observed between drought and flood years (Fig. 3A, B). The differences in BOD and COD concentrations were almost insignificant in the reservoir. These findings suggest low non-biodegradable organic matter in the reservoir. TN did not differ between the drought and flood years, and there was no significant N dilution during the Mon period, unlike other lakes in Korea [58]. TP increased significantly more (maximum = 91 μg/L) in flood years than in drought years (maximum = 56 μg/L) (Fig. 4A, B). The TN:TP ratio was higher in flood years during the Pre than Post periods, primarily attributed to increased TN (Fig. 4A, B). CHL-a increased during the Post in flood years, which is attributed to washout during the Mon [14]. The maximum value (61.5 μg/L) was observed in September, indicating the occurrence of an algal bloom. SS had the highest average value in September during a flood year, which was also attributed to an algal bloom (Fig. 4A, B). By contrast, SD decreased in July and August and increased in September during flood years, which is opposite to drought years. This suggests that inorganic SS dominates during the Mon [29], while organic matter algal blooms occur during the Post [23,31].

Similar results were observed for TN:TP, EC, CHL-a, SD, and CHL-a:TP in drought years, although no significant differences were found among the Pre, Mon, and Post (Table 2). However, among the three seasons, COD, TOC, TN, TP, WT, and SS differed significantly in drought years (p < 0.05). Post hoc analysis revealed that COD was significantly lower in the Pre than Mon (F = 5.201, p = 0.009), and TOC differed significantly between the Pre and Post (F = 4.832, p = 0.014), with higher values observed in the Post. TN and TP were significantly lower in the Pre than in both the Mon and Post (p < 0.01, Table 2). Similarly, significant differences were found in flood years for TOC, TP, TN:TP ratio, WT, CHL-a, and SD. Post hoc analysis revealed that TOC was significantly lower in the Pre than Post (F = 4.211, p < 0.05), and TP was significantly lower in the Pre than Post (F = 4.52, p < 0.05). The TN:TP ratio was significantly lower in Post than Mon (F = 4.011, p < 0.05), and WT was significantly higher in the Mon than Pre (F = 22.41, p < 0.001). Unlike drought years, CHL-a differed between the Pre and Post in flood years (F = 4.898, p < 0.05), with a unimodal peak in algal biomass in the Post. This finding is supported by previous studies of Korean reservoirs [17,31]. SD was significantly higher in the Pre than Mon (F = 4.23, p = 0.02). The CHL-a:TP ratio had a higher mean square value (0.4848) in flood years, indicating that CHL-a is formed in proportion to the amount of P supplied [29].

**Table 2:** One-way ANOVA test for drought and flood years (a: premonsoon (Pre), b: monsoon (Mon), c: postmonsoon (Post), Dr: Drought years, and Fl: Flood years).

Year	Parameters	Seasonal averages			Variance test					Post-hoc Comparison
		Pre (a)	Mon (b)	Post (c)	SS	ν	MS	*F*-value	*p*-value	Tukey test
Dr	BOD	1.09	1.32	1.19	0.027	2	0.013	0.763	0.472	a = b = c
	COD	2.64	3.18	3.04	0.057	2	0.029	5.201	**0.009**^b^	a < b (b = c)
	TOC	1.88	2.29	2.13	0.043	2	0.022	4.832	**0.014**^c^	a < c (a = b, c = b)
	TN	0.62	1.03	0.83	0.43	2	0.215	9.72	**0.0003**^a^	a < b (b = c)
	TP	15.2	26.7	20.7	0.407	2	0.203	6.673	**0.003**^b^	a < b (b = c)
	TN:TP	43.1	47.1	42.1	0.11	2	0.055	1.666	0.199	a = b = c
	WT	15.1	19.9	19.5	0.166	2	0.083	12.38	< **0.001**^a^	a < b (b = c)
	EC	128	126	95	0.102	2	0.051	1.431	0.248	a = b = c
	CHL-a	3.46	5.40	6.54	0.399	2	0.19	2.586	0.085	a = b = c
	SS	1.70	2.66	2.90	0.353	2	0.177	5.717	**0.006**^b^	a < b (b = c)
	SD	2.50	3.04	2.46	0.097	2	0.049	2.535	0.089	a = b = c
	CHL-a:TP	0.243	0.236	0.327	0.204	2	0.102	1.32	0.276	a = b = c
Fl	BOD	1.13	1.18	1.13	0.008	2	0.004	0.153	0.858	a = b = c
	COD	2.74	3.09	3.02	0.023	2	0.011	2.784	0.071	a = b = c
	TOC	1.65	2.15	1.94	0.049	2	0.025	4.211	**0.029**^c^	a < c (a = b, c = b)
	TN	0.96	1.00	0.93	0.016	2	0.008	0.515	0.601	a = b = c
	TP	17.7	26.1	24.8	0.52	2	0.265	4.52	**0.016**^c^	a < c (a = b, c = b)
	TN:TP	83.4	43.9	44.3	0.446	2	0.223	4.011	**0.024**^c^	c < b (a = b, a = c)
	WT	15.3	20.6	20.7	0.222	2	0.111	22.41	< **0.001**^a^	a < b (b = c)
	EC	89	105	83	0.071	2	0.036	1.89	0.162	a = b = c
	CHL-a	4.46	7.10	20.2	2.332	2	1.166	4.898	**0.011**^c^	a < c (a = b, c = b)
	SS	2.83	3.61	5.41	0.654	2	0.327	3.018	0.058	a = b = c
	SD	3.13	1.89	2.09	0.391	2	0.196	4.23	**0.02**^c^	b < a (a = c, b = c)
	CHL-a:TP	0.221	0.305	0.718	0.97	2	0.485	3.062	0.056	a < c (a = b, c = b)

SS: sum of squares; ν: degrees of freedom; MS – mean square; Significance level.

a Very strong.

b Strong.

c Moderate.

### 3.3 Relationship among water quality variables

**Fig. 5:**
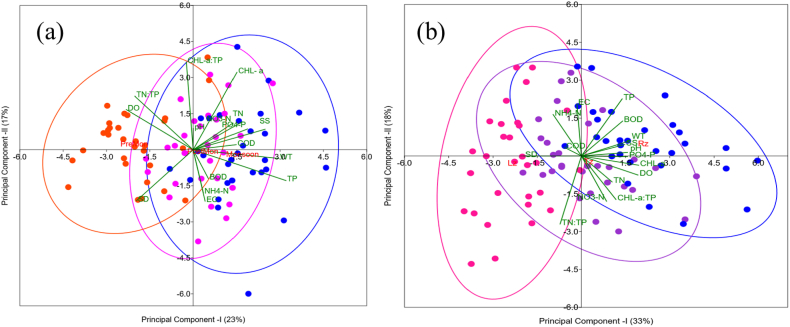
Principal component analysis (PCA) of the reservoir studied during 1993–2022; (a) seasonal biplot of PCA (red = premonsoon, blue = monsoon, and pink = postmonsoon), (b) longitudinal zonal biplot of PCA (pink = Lz, purple = Tz, and blue = Rz).

The multivariate PCA showed the monsoon seasonal and longitudinal fluctuations in water quality indicators with their correlations (Fig. 5). The analysis method simplifies the handling of large datasets with multiple parameters by converting the original variables into smaller, independent ones. It can also identify the main sources of pollution that affect water quality [59,64]. This study used the first two principal components (PC I and PC II), which were selected based on criteria of an eigenvalue greater than 1, to illustrate seasonal and longitudinal patterns. Seasonal PCs accounted for 40.65 % of the total variance, with PC I explaining 23.19 % and PC II explaining 17.46 % (Fig. 5a). PC I had strong positive loadings for WT and TP and a moderate positive loading for SS. However, it also had a moderate negative loading of TN:TP. The variation based on these loadings was statistically acceptable between the Pre and Post according to 95 % confidence seasonal ellipses. The outcome suggests that seasonal increases in suspended solids and phosphorus contents were in the system due to summer monsoon, which led to fluctuations in nutrient ratio [12,14]. Additionally, PC II had strong positive loadings of CHL-a and CHL-a:TP, and a moderate negative loading of SD, indicating specifically that increases in algal biomass negatively correlated with water transparency.

In addition to seasonal monsoon patterns, spatial PCA indicated the longitudinal variations in water quality indicators along the reservoir, accounting for 51.32 % of the total variance (Fig. 5b). PC I explained 33.22 % of the variance. It had strong positive WT and CHL-a and moderate positive loadings of SS, BOD, pH, TP, and PO_4_–P, suggesting longitudinal increases of the parameters from Lz to Rz. By contrast, SD exhibited strong negative loading. PC II accounted for 18.10 % of the variance and had moderate positive EC, TP, and NH_4_–N loadings. It also had TN:TP had strong negative loading of TN:TP and moderate negative loadings of CHL-a:TP and NO_3_–N. The multivariate statistic results supported our recent findings on the longitudinal analysis and were consistent with previous studies for Korean reservoirs [10,65].

### 3.4 Empirical models of reservoir algal biomass and water transparency

**Fig. 6:**
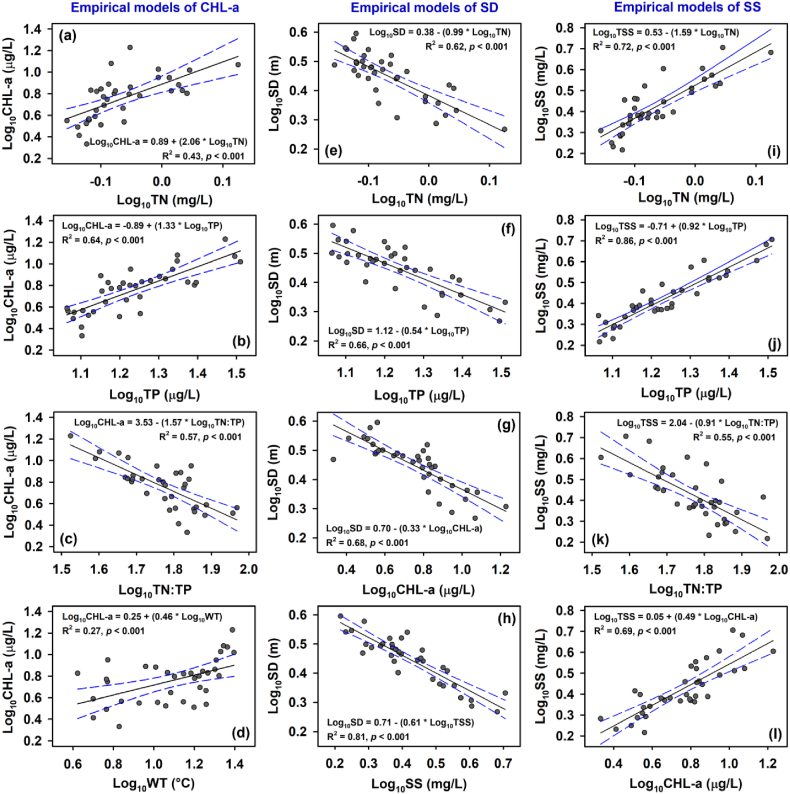
Empirical log-linear models of algal CHL-a (a–d), water transparency as measured by SD (e–h), and suspended solids (SS; i – l) based on total nitrogen (TN), total phosphorus (TP), TN:TP ratio, and water temperature (WT).

Empirical models based on simple linear regression showed that phosphorus was the most significant determinant for the variation in longitudinal-monthly algal CHL-a averages (n = 36) for 30 years, closely followed by TN:TP ratio (Fig. 6a–d). TP and TN:TP explained approximately 64 % and 57 % of the variation (*p* < 0.001) according to the coefficient determination (R^2^). TN:TP showed a strong negative correlation with TP (R^2^ = 0.72, *p* < 0.001), but it had a weak negative correlation with TN (R^2^ = 0.25, *p* < 0.01). These correlations highlighted the importance of phosphorus availability relative to nitrogen in shaping algal growth dynamics, suggesting a phosphorus limitation within the system. This inference aligns with earlier empirical studies on Korean multipurpose and deep reservoirs [10,11,65]. Additionally, TN and WT significantly and positively correlated with CHL-a (*p* < 0.001), with respective R^2^ values of 0.43 and 0.21 (Fig. 6a–d), indicating their influence on algal biomass. Therefore, we developed multiple linear regression models (MLR) using WT, TN, and TN:TP added on TP (Table S1). The best MLR was based on the interplay between TP, TN:TP, and WT and accounted for 67 % of the CHL-a variation, indicating a slight improvement in prediction accuracy as measured by R^2^.

Moreover, when SD was empirically modeled based on nutrients (TN and TP), CHL-a, and SS (Fig. 6e–h), SS accounted for a substantial part (81 %) of the variation in SD (Fig. 6h). This suggests that SS was the dominant regulator of water transparency and underwater light availability, due to its strong association with increased nutrients (TN and TP) and CHL-a levels (Fig. 6i–l). Notably, SS showed a particularly robust correlation with TP (R^2^ = 0.86, *p* < 0.001). As previously discussed by Refs. [23,66], suspended solids tend to bind efficiently and transport phosphorus particles within the water column during the summer monsoon, especially in high concentrations, influencing SD variations. Consequently, the fluctuations in SD may be more attributable to inorganic suspended matter from monsoon runoffs rather than algal biomass. It aligns with the findings of [30], who observed a greater predictive power of SS over CHL-a in determining SD using machine learning techniques. Hence, employing SD as a metric for assessing trophic states in Korean reservoirs may be misleading, particularly considering the influence of monsoon-specific conditions. Similar observations have been reported in other regions, such as the Eastern Chinese Plains [18] and the Mediterranean [67], where reductions in SD were primarily attributed to inorganic turbidity and matter rather than CHL-a concentrations. Future studies should address the challenge of SD in trophic state assessment.

However, the interactive correlations among nutrients, suspended solids, SD, and CHL-a levels can underscore the dynamics of nutrient loading and its implications for algal growth and water transparency in the studied reservoir system. Elevated phosphorus levels, facilitated by suspended solids, can contribute to heightened CHL-a levels. As algal biomass increases, it can further diminish water transparency, impacting light penetration and potentially establishing a feedback loop where heightened algal biomass perpetuates decreased water transparency [61], exacerbating the situation.

### 3.5 Monsoon-seasonal analyses of TSI and TSID along the system with the influence of drought and flood years regimes

**Fig. 7:**
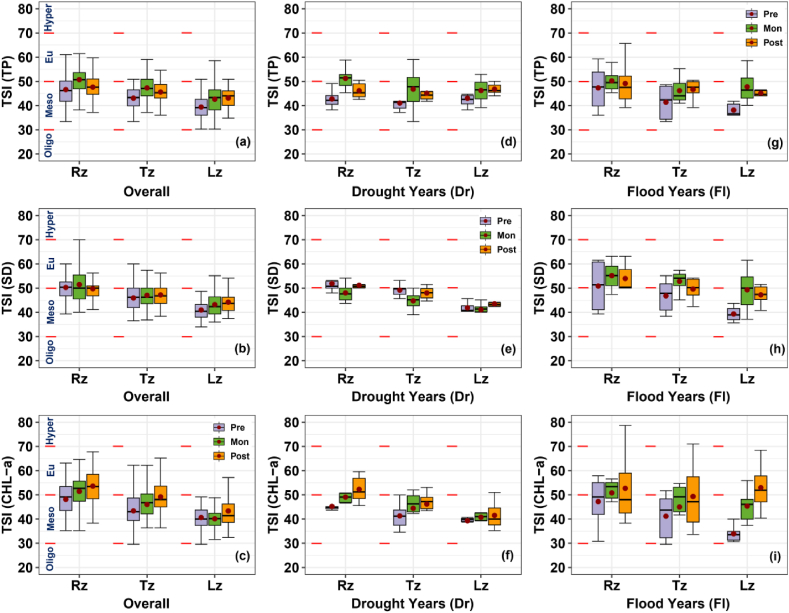
Longitudinal and monsoon-seasonal variations in trophic state indices based on total phosphorus (TSI (TP)), Secchi depth (TSI (SD)), and CHL-a (TSI (CHL-a) for overall (a–c), drought, and flood years (d–i). Seasons were categorized as premonsoon (Pre), monsoon (Mon), and postmonsoon (Post). The trophic status was classified as oligotrophic (Oligo), mesotrophic (Meso), eutrophic (Eu), and hypereutrophic (Hyper).

Assessing reservoir trophic status is crucial for its ecological health [11]. In this regard, a longitudinal and monsoon-seasonal analysis was conducted based on the TSIs of TP, SD, and CHL-a following Carlson's method [24]. The results revealed that the reservoir trophic status ranged from mesotrophic to eutrophic overall and varied between drought and flood years (Fig. 7).

TSI (TP) averages decreased longitudinally from Rz to Lz, with seasonal increases being evident in Mon at each zone (Fig. 7a). The overall TSI (SD) pattern was similar to that of TSI (TP) (Fig. 7b), and the seasonal increases in both indices were primarily attributed to heightened inorganic suspended solids carried with phosphorus due to monsoon runoff into the reservoir [23,29]. Also, a noticeable longitudinal decrease was observed in the TSI (CHL-a) averages from Rz to Lz (Fig. 7c). Seasonal increases were evident in both Mon and Post at Rz and Lz. Particularly, eutrophic states were observed in Rz during Mon and Post, indicating a heightened risk of algal blooms. Moreover, TSI (CHL-a) increased at Lz during the Post, with 75 % of its distribution pointing towards a eutrophic state (Fig. 7c).

Our analysis also revealed a crucial aspect regarding the impact of monsoon intensity on trophic state conditions, which varied significantly between flood and drought years (Fig. 7d–i), particularly for TSI (SD) and TSI (CHL-a). The variability in these indices for each season was considerably low in drought years (Fig. 7e and f). However, this variability increased during flood years (Fig. 7h and i). The TSI (SD) averages were notably higher in Mon with the eutrophic state during flood years (Fig. 7e) than in drought years (Fig. 7f). Furthermore, TSI (CHL-a) averages demonstrated an observable increase in eutrophication risk in Post after intense monsoon periods (Fig. 7i), particularly in Lz. In drought years, there were longitudinal decreases in TSI (CHL-a) averages for each season (Fig. 7f). However, during flood years, the spatial variation was interrupted, and all zones experienced eutrophic states in Post. The findings supported that intense rainfall events would trigger algal blooms in subsequent months, particularly in the lacustrine site [31].

**Fig. 8:**
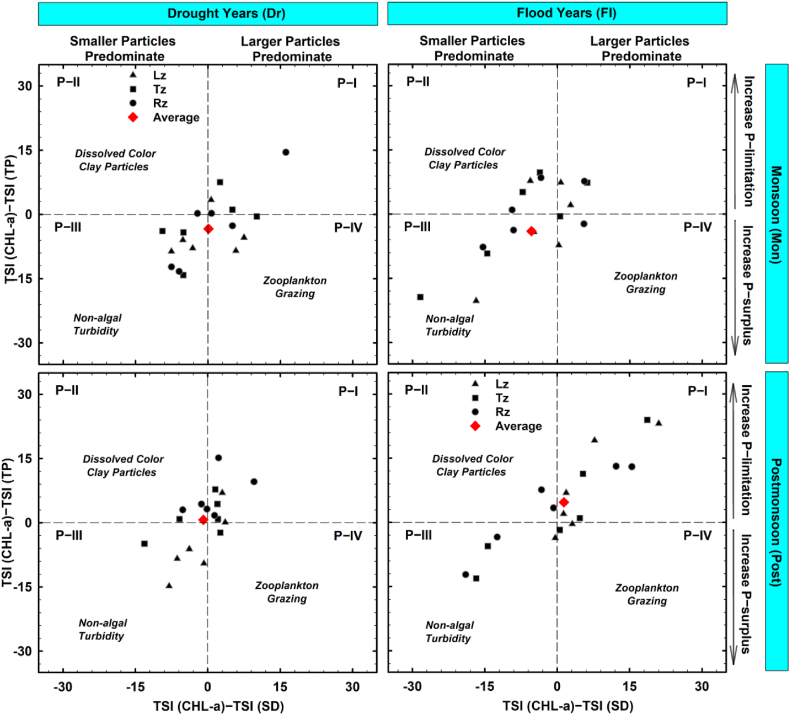
Variations in trophic state index deviations during monsoon and postmonsoon seasons between drought and flood years.

TSID analyses, furthermore, suggested that phosphorus availability strongly influences the reservoir's trophic status, with several episodic impacts of non-algal turbidity at each site during Mon (Fig. S2), according to Ref. [19]. The findings were aligned with the insights obtained by empirical models of CHL-a and SD, supporting a P-limitation scenario in the temperate deep system along with the seasonal conditions of the summer monsoon [68]. Also, increases in non-algal turbidity influence (P-III) were evident during Mon in flood years compared to drought years, as indicated by mean values of TSID (Fig. 8). TSID also showed algal bloom occurrences (P–I) were more prevalent during Post in flood years compared to drought years.

### 3.6 Long-term trend analysis

**Fig. 9:**
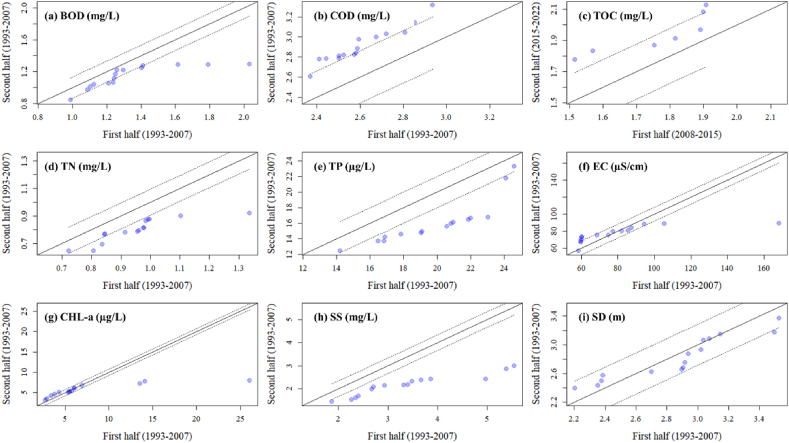
Innovative trend analysis (ITA) of water quality indicators over the study period, including biological oxygen demand (BOD), chemical oxygen demand (COD), total organic carbon (TOC), total nitrogen (TN), total phosphorus (TP), electric conductivity (EC), algal CHL-a, suspended solids (SS), and Secchi depth (SD).

Our extensive dataset, covering a 30-year span, provided a platform for investigating the long-term trends in water quality indicators using the MK test and ITA. The analysis of yearly reservoir means revealed notable overall trends: BOD, TN, TP, TSS, and SD showed decreasing trends, whereas COD showed an increasing trend according to the MK test (Table 3). Conversely, TOC, EC, and CHL-a did not exhibit significant trends over the studied period. The ITA corroborated the findings, except for BOD and SD (Fig. 9a–i).

**Table 3:** Long-term trend analysis of water quality indicators in the reservoir over the 30-year period (1993–2022).

Indicators	S	τ	Slope	Trend
BOD	**−176**	**−0.405**	**−0.014**^b^	**Decreasing**
COD	**240**	**0.552**	**0.019**^a^	**Increasing**
TOC	39	−0.371	0.019	No trend
TN	**−145**	**−0.333**	**−0.007**^c^	**Decreasing**
TP	**−156**	**−0.359**	**−0.194**^b^	**Decreasing**
EC	35	0.08	0.137	No trend
CHL-a	27	0.062	0.024	No trend
TSS	**−117**	**−0.269**	**−0.038**^c^	**Decreasing**
SD	**−66**	**−0.152**	**−0.010**^b^	**Decreasing**

a *p* < 0.001.

b *p* < 0.01.

c *p* < 0.05.

The contrasting trends observed between BOD and COD suggest a potential shift in the pollutants, possibly indicating an increase in the proportion of non-biodegradable organic matter [65]. While the decline in nutrients and suspended solids over the long-term period might suggest an improvement in reservoir water quality, it is important to consider other factors, such as increased water volume and storage capacity, which could also contribute to these changes. Early and recent studies [[38], [39], [40]] underscore the persistent influence of intense non-point pollution sources on reservoir water quality, potentially fostering algal blooms. Interannual-seasonal variations in hydrological conditions and nutrient availability can regulate the long-term dynamics of algal biomass during growing seasons, mainly triggered by the summer monsoon [10,59]. Monsoon-specific conditions might lead to no significant trend in algal CHL-a levels in the system studied. Also, diffuse nutrient losses worldwide are considered the main drivers of eutrophication [69]. This highlights the ongoing relevance and significance of monitoring and controlling the impacts of non-pollution sources on the system and non-biodegradable pollutants, particularly by considering the summer monsoon intensity.

### 3.7 Predictions of algal CHL-a and SD based on machine learning approach

**Fig. 10:**
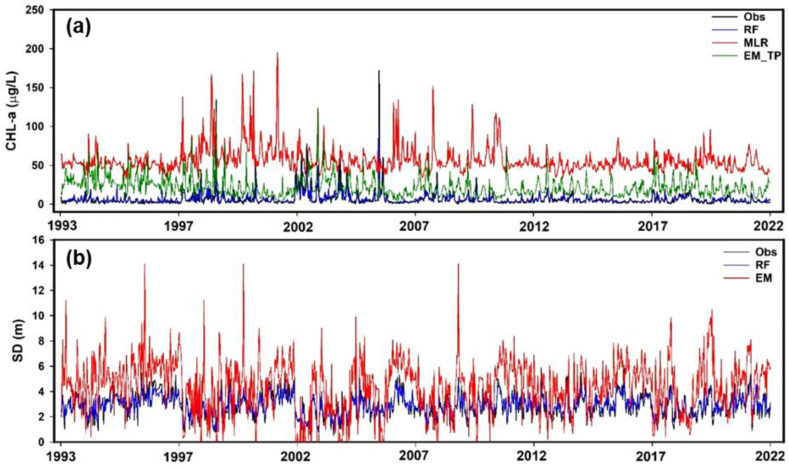
The comparison of (a) predicted and observed CHL-a values based on the best multiple linear regression (MLR), empirical model (EM_TP), and random forest (RF), along with (b) SD predictions by RF and EM.

The study also analyzed the modeling accuracies of the random forests (RF) machine learning, MLR, and EM models in predicting CHL-a and SD levels in the Juam Reservoir. The time series plots clearly illustrated the observed and predicted levels of both CHL-a and SD, generated using RF, MLR, and Ems (Fig. 10a and b). CHL-a values predicted by the RF model closely aligned with the observed values, indicating superior performance compared to predictions generated by both the best MLR and EM models. To validate this qualitative assessment, the study conducted a rigorous analysis utilizing the coefficient of determination (R^2^) to compare the modeling accuracies of the three models. The RF model demonstrated a significantly higher R^2^ value of 0.72, surpassing the performance of both the MLR and EM models. When employing the RF model, this elevated R^2^ value indicates a robust correlation and low error between the predicted and observed CHL-a levels, reaffirming its efficacy in modeling CHL-a dynamics within the Juam Reservoir. Based on these findings, the study recommends using the RF model to predict algal biomass and water transparency in multipurpose reservoirs.

## 4 Conclusions

This study provides important insights into the longitudinal and monsoon seasonal variation in water chemistry in the Asian temperate reservoir, highlighting its impact on algal biomass as indicated by CHL-a and long-term trends over three decades. The study used various analytical methods, including multivariate analysis, empirical models, trophic state analysis, and machine learning approaches. The findings revealed longitudinal heterogeneity in nutrients, organic matter, CHL-a, and SS along the Rz, Tz, and Lz. Seasonal nutrient and suspended solid fluctuations and decreased water transparency were observed during the summer monsoon (Mon), influenced by external inputs and varying dynamics during flood and drought years. Furthermore, we found a monomodal increase in CHL-a levels after the summer monsoons, particularly during intense rainfall, indicating successful algal growth during the post-monsoon period.

According to empirical models, phosphorus was found to be the key determinant of algal biomass, while suspended solids played a significant role in regulating water transparency. TSID analyses also suggested that phosphorus availability strongly influences the reservoir's trophic status, with episodes of non-algal turbidity at each site during the monsoons. The findings supported a P-limitation scenario in the temperate deep system along with the seasonal conditions of the summer monsoon.

Our study also highlighted overall trends in water quality parameters over the long period, albeit with the signaling of the pollutants towards non-biodegradable organic matter. Moreover, while declining nutrient and suspended solid levels could indicate improved water quality, the persistent influence of non-point pollution sources underscores the need for continued monitoring and control, particularly considering seasonal variations and the impact of the summer monsoon on algal biomass dynamics and nutrient loadings. The predictive capabilities demonstrated by random forests machine learning suggested the potential utility of such an approach in forecasting algal biomass and water transparency.

## Data availability statement

Data will be made available on reasonable request.

## CRediT authorship contribution statement

**Sang-Hyeon Jin:** Writing – original draft, Formal analysis. **Namsrai Jargal:** Writing – review & editing, Writing – original draft, Formal analysis. **Thet Thet Khaing:** Writing – original draft, Formal analysis. **Min Jae Cho:** Writing – original draft, Formal analysis, Data curation. **Hyeji Choi:** Writing – original draft, Formal analysis. **Bilguun Ariunbold:** Writing – original draft, Formal analysis. **Mnyagatwa Geofrey Donat:** Writing – original draft, Formal analysis. **Haechan Yoo:** Writing – original draft, Formal analysis. **Md Mamun:** Validation, Formal analysis. **Kwang-Guk An:** Writing – review & editing, Validation, Supervision, Resources, Funding acquisition, Data curation.

## Declaration of competing interest

The authors declare that they have no known competing financial interests or personal relationships that could have appeared to influence the work reported in this paper.
