# Supplementary material for: Long-term prediction of algal chlorophyll based on empirical models and the machine learning approach in relation to trophic variation in Juam Reservoir, Korea
Source: Heliyon. 2024 May 28;10(11):e31643. doi: 10.1016/j.heliyon.2024.e31643 (PMC11176781; doi:10.1016/j.heliyon.2024.e31643)
Supplement: Multimedia component 1 [file mmc1.docx]

**SUPPLEMENTARY MATERIALS**

**Long-term Prediction of Algal Chlorophyll Based on Empirical Models and a Machine Learning Approach in Relation to Trophic Variation in the Juam Reservoir, Korea**

Sang-Hyeon Jin^a^, Namsrai Jargal^a^, Thet Thet Khaing^a^, Min Jae Cho^a^, Hyeji Choi^a^, Bilguun Ariunbold^a^, Mnyagatwa Geofrey Donat^a^, Haechan Yoo^a^, Mamun Md^a, b^, Kwang-Guk An^a^ *****

^a^ Department of Bioscience and Biotechnology, Chungnam National University, Daejeon 34134, Republic of Korea

^b^ Department of Earth Sciences, Southern Methodist University, Dallas, TX 75205, United States of America

* Corresponding author: E-mail address: [kgan@cnu.ac.kr](mailto:kgan@cnu.ac.kr) (K-G. An).


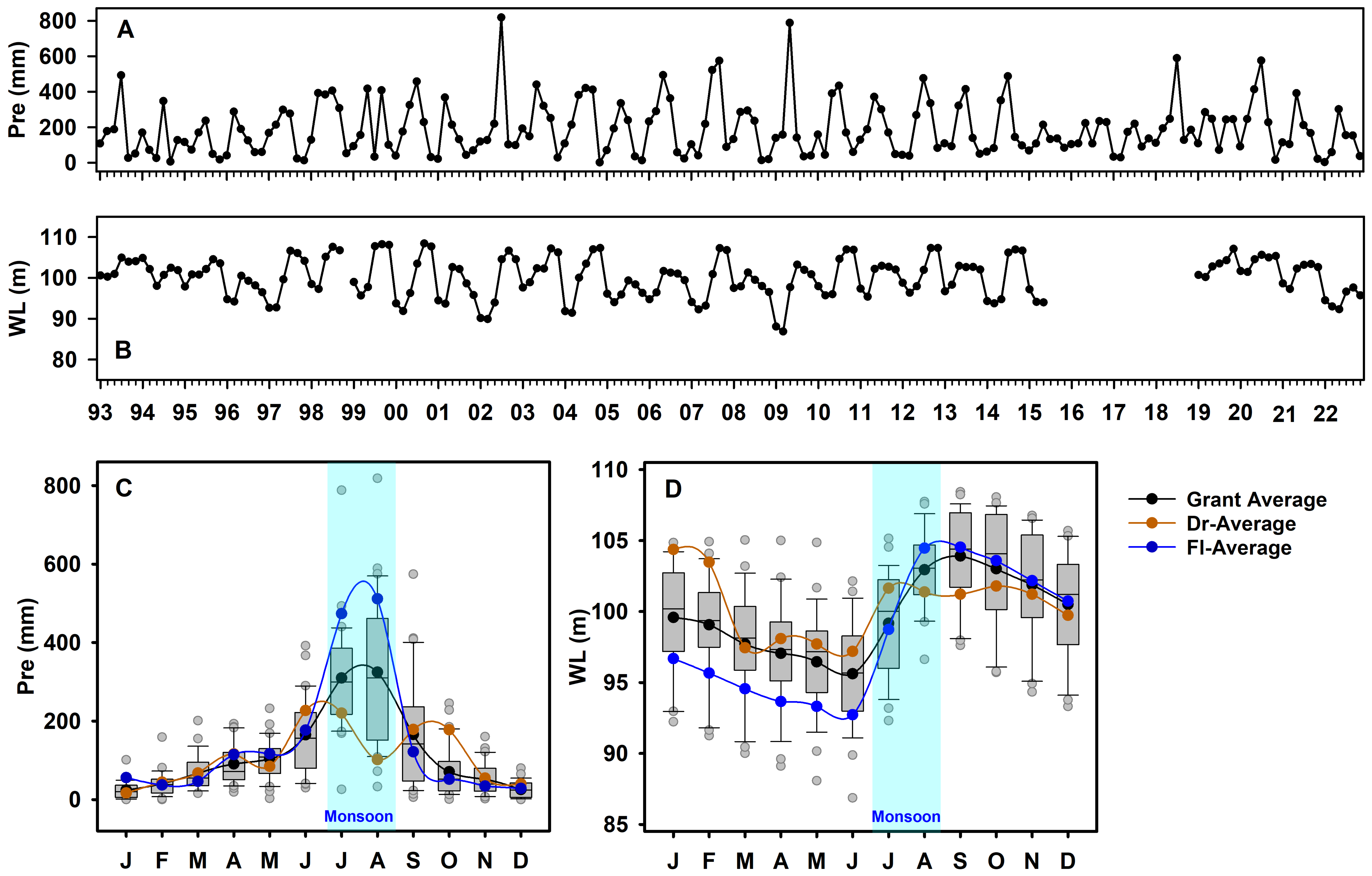


**Fig. S1.** Long-term monsoon-seasonal dynamics of (**A**) precipitation [Pre] and (**B**) water level [WL] from 1993 to 2022 and their overall monthly dynamics with the comparison of drought and flood years (**C** and **D**).





**Fig. S2.** Trophic state index deviation (TSID) analysis on monsoon-seasonal values of trophic state index based on TP, SD, and CHL-a at each zone.
